# Circulating Non-Coding RNA Levels Are Altered in Autosomal Dominant Frontotemporal Dementia

**DOI:** 10.3390/ijms232314723

**Published:** 2022-11-25

**Authors:** Chiara Fenoglio, Maria Serpente, Caterina Visconte, Marina Arcaro, Federica Sorrentino, Marianna D’Anca, Andrea Arighi, Emanuela Rotondo, Roberto Vimercati, Giacomina Rossi, Elio Scarpini, Daniela Galimberti

**Affiliations:** 1 Department of Pathophysiology and Transplantation, Dino Ferrari Center, University of Milan, 20122 Milan, Italy; 2 Fondazione, IRCCS Ca’ Granda, Ospedale Maggiore Policlinico, 20122 Milan, Italy; 3 Department of Biomedical, Surgical and Dental Sciences, Dino Ferrari Center, University of Milan, 20122 Milan, Italy; 4 Unit of Neurology V—Neuropathology, Fondazione IRCCS Istituto Neurologico Carlo Besta, 20133 Milan, Italy

**Keywords:** frontotemporal disease, microRNA, long non-coding RNA, *GRN*, *MAPT*, *C9ORF72*, TDP-43, tau

## Abstract

Frontotemporal Dementia (FTD) represents a highly heritable neurodegenerative disorder. Most of the heritability is caused by autosomal dominant mutations in the Microtubule-Associated Protein Tau (*MAPT*), Progranulin (*GRN*), and the pathologic exanucleotide expansion of *C9ORF72* genes. At the pathological level, either the tau or the TAR DNA-binding protein (TDP-43) account for almost all cases of FTD. Pathogenic mechanisms are just arising, and the emerging role of non-coding RNAs (ncRNAs), such as microRNAs (miRNA) and long non-coding RNAs (lncRNAs), have become increasingly evident. Using specific arrays, an exploratory analysis testing the expression levels of 84 miRNAs and 84 lncRNAs has been performed in a population consisting of 24 genetic FTD patients (eight *GRN*, eight *C9ORF72*, and eight *MAPT* mutation carriers), eight sporadic FTD patients, and eight healthy controls. The results showed a generalized ncRNA downregulation in patients carrying *GRN* and *C9ORF72* when compared with the controls, with statistically significant results for the following miRNAs: miR-155-5p (Fold Change FC: 0.45, *p* = 0.037 FDR = 0.52), miR-15a-5p (FC: 0.13, *p* = 0.027, FDR = 1), miR-222-3p (FC: 0.13, *p* = 0.027, FDR = 0.778), miR-140-3p (FC: 0.096, *p* = 0.034, FRD = 0.593), miR-106b-5p (FC: 0.13, *p* = 0.02, FDR = 0.584) and an upregulation solely for miR-124-3p (FC: 2.1, *p* = 0.01, FDR = 0.893). Conversely, *MAPT* mutation carriers showed a generalized robust upregulation in several ncRNAs, specifically for miR-222-3p (FC: 22.3, *p* = 7 × 10^−6^, FDR = 0.117), miR-15a-5p (FC: 30.2, *p* = 0.008, FDR = 0.145), miR-27a-3p (FC: 27.8, *p* = 6 × 10^−6^, FDR = 0.0005), miR-223-3p (FC: 18.9, *p* = 0.005, FDR = 0.117), and miR-16-5p (FC: 10.9, *p* = 5.26 × 10^−5^, FDR = 0.001). These results suggest a clear, distinctive pattern of dysregulation among ncRNAs and specific enrichment gene pathways between mutations associated with the TDP-43 and tau pathologies. Nevertheless, these preliminary results need to be confirmed in a larger independent cohort.

## 1. Introduction

Frontotemporal dementia (FTD) represents the most common cause of presenile dementia, usually affecting people under 60 years old [1]. Clinically, patients present with changes in either behavior or personality. Up to 40% of patients have a history of familial transmission, with nearly 10% of patients showing an autosomal dominant inheritance pattern [1]. The majority of familial FTD patients carry mutations in the Microtubule-Associated Protein Tau (*MAPT*) and Progranulin (*GRN*) genes, and the pathologic expansion of the hexanucleotide G_4_C_2_ repeats in the first intron of the *C9ORF72* gene [2]. At the pathological level, either the tau or the TAR DNA-binding protein, with a molecular weight of 43 kDA protein (TDP-43), account for almost all cases of frontotemporal lobar degeneration [3].

Due to clinical and pathologic heterogeneity, the genetic basis of FTD is complex, and the underlying mechanisms are just arising. The regulation of gene expression at transcriptional and post-transcriptional levels plays a pivotal role in the development and function of the Central Nervous System (CNS) [4]. In this scenario, the emerging role of non-coding RNAs (ncRNAs) implicated in the pathogenesis of neurodegenerative diseases, such as microRNAs (miRNA) and long non-coding RNAs (lncRNAs), has become increasingly evident [5]. Based on their size, ncRNAs should be categorized into small non-coding RNAs (<200 nt) and long non-coding RNAs (>200 nt). They are defined as single-stranded RNA molecules without a protein-coding function, but while miRNAs mainly induce repression or degradation on hundreds of different mRNAs, lncRNAs have heterogeneous interacting partners and modes of action. Their involvement in a wide range of biological and pathological processes has been documented [5,6]. The significant role of miRNAs in neurodegenerative diseases is now established, as several studies indicate that they have huge potential to regulate gene expression, as they target hundreds of protein-coding genes [7]. Because they are secreted into biological fluids and are stable in the bloodstream, ncRNAs represent interesting tools for the diagnosis and/or prognosis of diseases [8]. Indeed, there is evidence for the potential use of miRNAs as biomarkers to discover and discriminate neurodegenerative diseases [9,10]. While miRNAs’ potentialities in neurodegenerative diseases have been largely analyzed, the critical role of lncRNAs is still under investigation, despite their abundance in the CNS. Recently, some studies have shown the relevant role of lncRNAs in the development and functioning of CNS [11,12]. Their association with neurodegenerative disorders, such as Alzheimer’s disease (AD), Parkinson’s disease (PD), Amyotrophic Lateral Sclerosis (ALS), and others, is becoming evident (see for reviews [13,14]). Therefore, how lncRNAs are involved in these diseases needs to be further explored and deeply investigated. Furthermore, our knowledge of the lncRNAs’ involvement in FTD is scarce due to the poor quality of studies on this argument, especially those focused on miRNAs [15,16,17].

Circulating miRNAs have been proposed as promising biomarkers for neurodegenerative diseases, including FTD [18,19], whereas the role of circulating lncRNAs in FTD still constitutes a new area in which ncRNAs are under study as potential biomarkers.

Given these assumptions, and the different underlying pathologies in FTD patients, our study aimed to fill the gap between genetics, clinical phenotype, and biomarker discovery in order to discover the biological mechanisms at the basis of neuronal death in FTD patients carrying a pathogenic mutation versus non-carriers and controls.

To this purpose, an exploratory analysis, using specific arrays in order to profile cell-free circulating ncRNAs (miRNAs and lncRNAs), was performed in sporadic and autosomal dominant FTD caused by *GRN* and *C9ORF72* and *MAPT* mutations.

## 2. Results

The expression profile of the 84 miRNAs tested revealed several dysregulations in the serum of FTD patients. The statistically significant threshold was not reached for most of the investigated miRNAs after multiple comparison adjustment. Nevertheless, due to the exploratory design of the study, we considered the raw *p*-value for the analysis but reported also the FDR value. 

**Figure 1:**
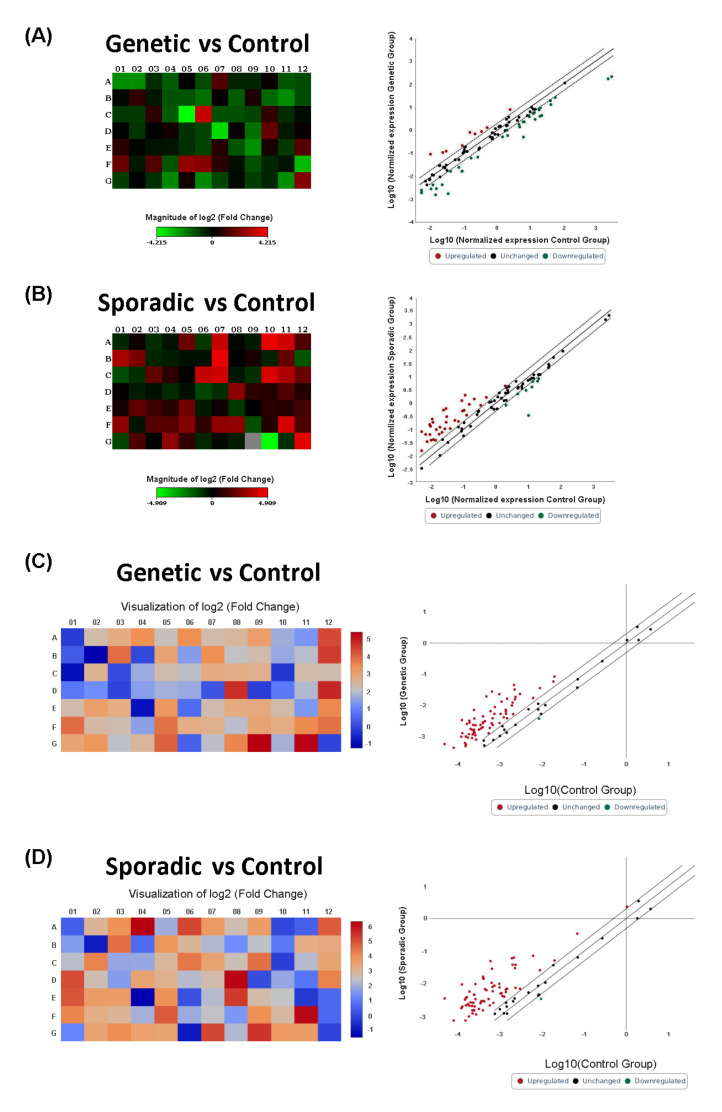
(**A**,**B**) Heat map and scatter plot of ncRNA profiling for genetic FTD patients and sporadic cases versus control group. Heat Maps: FTD genetic mutations carriers (**A**); sporadic cases (**B**). Data are expressed as fold change (FC). Each square represents one of the 84 miRNAs in the array. Blue indicates downregulation, red upregulation. (**C**,**D**) Heat Map of the expression fold changes for the 84 lncRNAs array in genetic FTD carriers (**C**); sporadic FTD patients (**D**). Data are expressed as fold change (fold difference). Blue indicates downregulation, red upregulation.

In particular, a generalized downregulation was observed in the serum miRNAs expression from genetic FTD patients when compared to the controls, whereas an overall upregulation was observed for patients with sporadic FTD versus the controls (Figure 1A,B, Supplementary Tables S1 and S2).

Each scatter plot compares the normalized expression of each miRNA on the array between the two selected groups by plotting them against one another to quickly visualize large miRNA expression changes. The central line indicates an unchanged miRNA expression. The dotted lines indicate the selected fold regulation threshold. Data points beyond the dotted lines in the upper left and lower right sections meet the selected fold regulation threshold.

**Figure 2:**
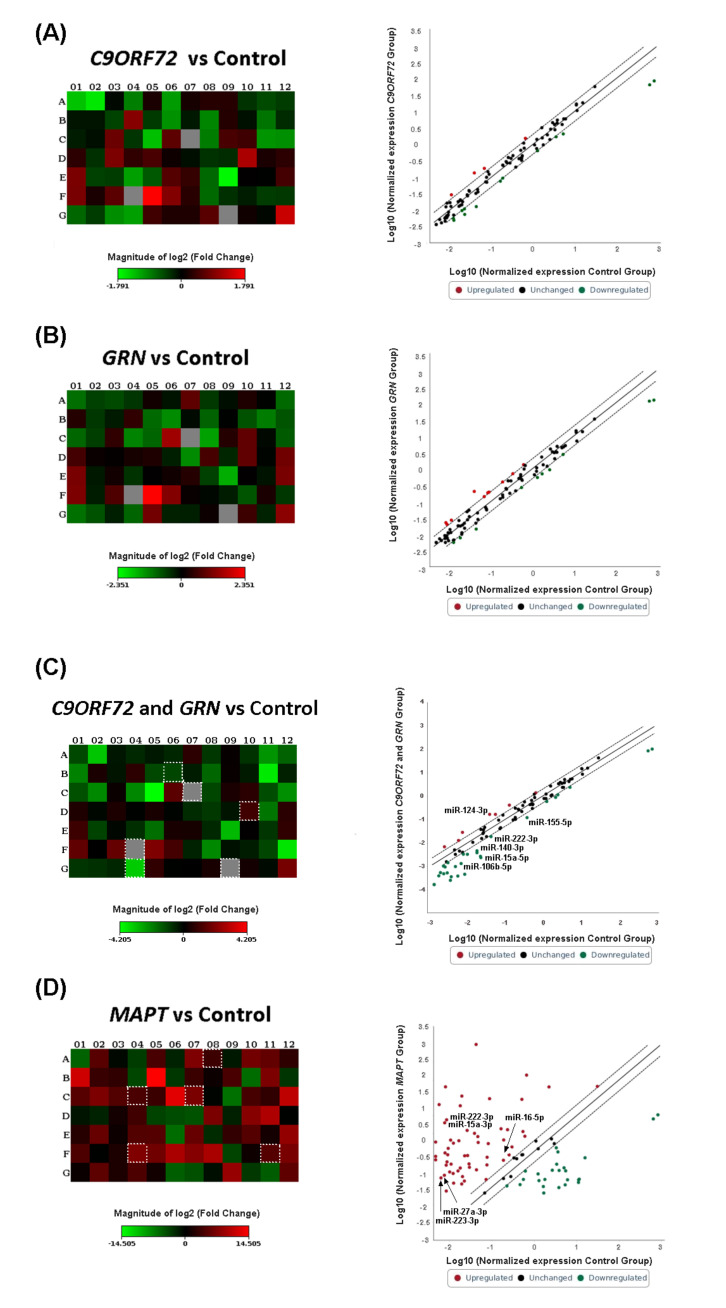
Heat map and scatter plot of miRNA profiling for FTD patients with *C9ORF72* expansion (**A**); *GRN* mutation (**B**); *GRN* + *C9ORF72* grouped mutation (**C**); *MAPT* mutation (**D**); versus control group. Heat Maps: FTD *C9ORF72* expansion carriers (**A**); *GRN* mutation carriers (**B**); *GRN* + *C9ORF72* grouped mutation (**C**); *MAPT* mutation carriers (**D**). Each square represents one of the 84 miRNAs in the array. Squares corresponding to the miRNAs described in the text are highlighted. Green indicates downregulation, red upregulation. Each scatter plot compares the normalized expression of every miRNA on the PCR array between the two selected groups by plotting them against one another to quickly visualize large expression changes. The central diagonal line indicates unchanged gene expression, while the outer diagonal lines indicate the selected fold regulation threshold. miRNAs with data points beyond the outer lines in the upper left and lower right corners are upregulated or downregulated, respectively, by more than the fold regulation threshold in the y-axis Group relative to the x-axis Group.

The trend to downregulation was maintained when *GRN* and *C9ORF72* carriers were considered singularly (Figure 2 panels A–B, Supplementary Tables S3 and S4) but were revealed to be stronger when the results were grouped according to the pathological protein deposition status (Figure 2, panel C, Supplementary Table S5). Thus, when patients carrying *GRN* and *C9ORF72* were grouped together for the analysis according to their underlying TDP-43 pathology, a trend to statistically significant altered levels in several miRNAs was shown. Specifically, miR-155-5p, miR-15a-5p, miR-222-3p, miR-140-3p, and miR-106b-5p were downregulated compared with the controls (FC 0.45 *p* = 0.037 *FDR* = 0.052, FC 0.128 *p* = 0.027 *FDR* = 1, FC 0.128 *p* = 0.027 *FDR* = 0.778, FC 0.096 *p* = 0.035 *FDR* = 0.593 and FC 0.128 *p* = 0.02 FDR = 0.584, respectively) whereas miR-124-3p appeared to be upregulated (FC: 2.089, *p* = 0.010 FDR = 0.893). Conversely, when *MAPT* mutation carriers were considered, a trend to predominant upregulation in miRNAs expression levels was found (Figure 2D). In particular, miR-222-3p, miR-15a-5p, miR-27a-3p, miR-223-3p, and miR-16-5p showed higher expression levels in *MAPT* mutation carriers compared with the controls (FC 22.3 *p* = 7 × 10^−6^ FDR= 5 × 10^−4^, FC 30.2 *p* = 0.008 FDR = 0.145, FC 27.8 *p* = 6 × 10^−6^ FDR= 5 × 10^−4^, FC 18.9 *p* = 0.005 FDR = 0.117 and FC 10.9 *p* = 5.2 × 10^−5^ FDR = 0.001, respectively).

In order to better elucidate the function of the best-dysregulated miRNAs, an in silico approach was used to identify potential relevant pathways for the disease. The publicly available bioinformatics tools that miRNet implemented with DisGeNET were employed for a KEGG analysis of target and pathway predictions. 

**Figure 3:**
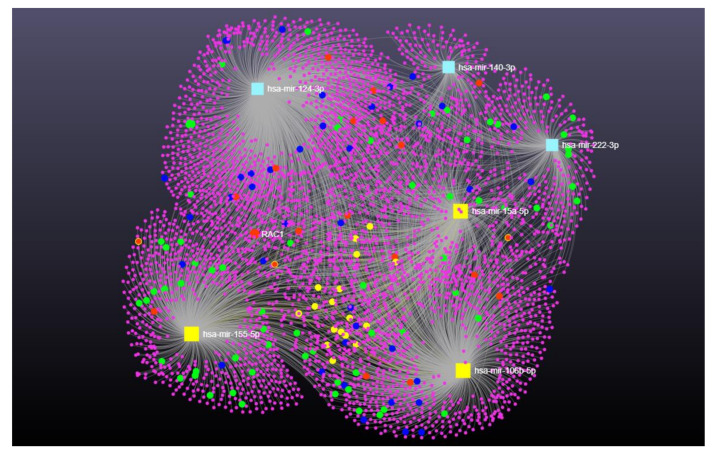
Network visualization for the enrichment analysis of miR-155-5p, miR-15a-5p, miR124-3p, miR-222-3p, miR-140-3p, and miR-106b-5p in *GRN* and *C9ORF72* FTD group. Yellow squares represent miRNAs, and pink circles are their targets. In particular, yellow circles represent the shared interactions. Phagosome and regulation of autophagy target genes are represented in red, axon guidance pathway in blue, and RNA degradation and transport are highlighted in green.

**Figure 4:**
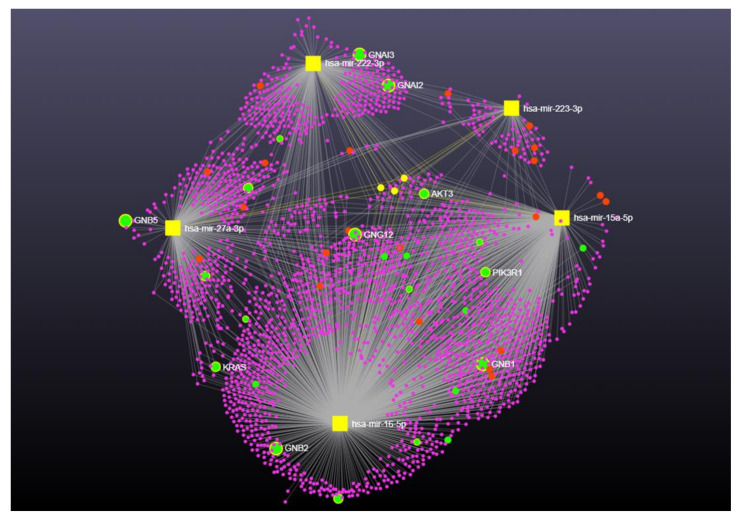
Network visualization for the enrichment analysis of miR-223-3p, miR-15a-5p, miR-222-3p, and miR-16-5p in *GRN* and *MAPT* FTD group. Yellow squares represent miRNAs, and pink circles are their targets. In particular, yellow circles represent the shared interactions between miR-155-5p, miR-106b-5p, and miR-15a-5p. Chemokines signaling target genes are represented in red, whereas Synaptic vesicle cycle, GABAergic synapse, and cholinergic synapse target genes are highlighted in green.

A visual exploration of the network analysis results is shown in Figure 3 and Figure 4. MiRNet analysis revealed that the best hits miRNAs for patients carrying *GRN* and *C9ORF72* mutations targeted 3947 genes. They do not appear to show common target genes; however, there are some shared interactions in which only miR-15a-5p, miR-106b-5p, and miR-155-5p are grouped together (Figure 3, yellow circles).

The results of functional enrichment using the KEGG database revealed some pathways significantly enriched in *GRN* and *C9ORF72* patients, such as phagosome (*p* = 0.0167, FDR = 0.0251), regulation of autophagy (*p* = 0.0182, FDR = 0.0251), RNA degradation (*p* = 0.0244, FDR = 0.0377), RNA transport (*p* = 0.0247, FDR = 0.0246), and axon guidance (*p* = 0.00806, FRD = 0.0161) pathways (Figure 3, Supplementary Tables S7 and S8).

When the best hits miRNAs raised from the *MAPT* patient group were considered for the functional enrichment analysis, 2301 target genes were detected. Few genes were reported for shared interaction (Figure 4, yellow circles). The functional enrichment analysis showed a trend to enrichment for chemokine signalling (*p* = 0.0573, FDR = 0.0749), synaptic vesicle cycle (*p* = 0.0636, FDR = 0.0749), GABAergic synapse (*p* = 0.0738, FDR = 0.0749), and cholinergic synapse (*p* = 0.0745, FDR = 0.0749) pathways (Figure 4, Supplementary Tables S9 and S10).

**Figure 5:**
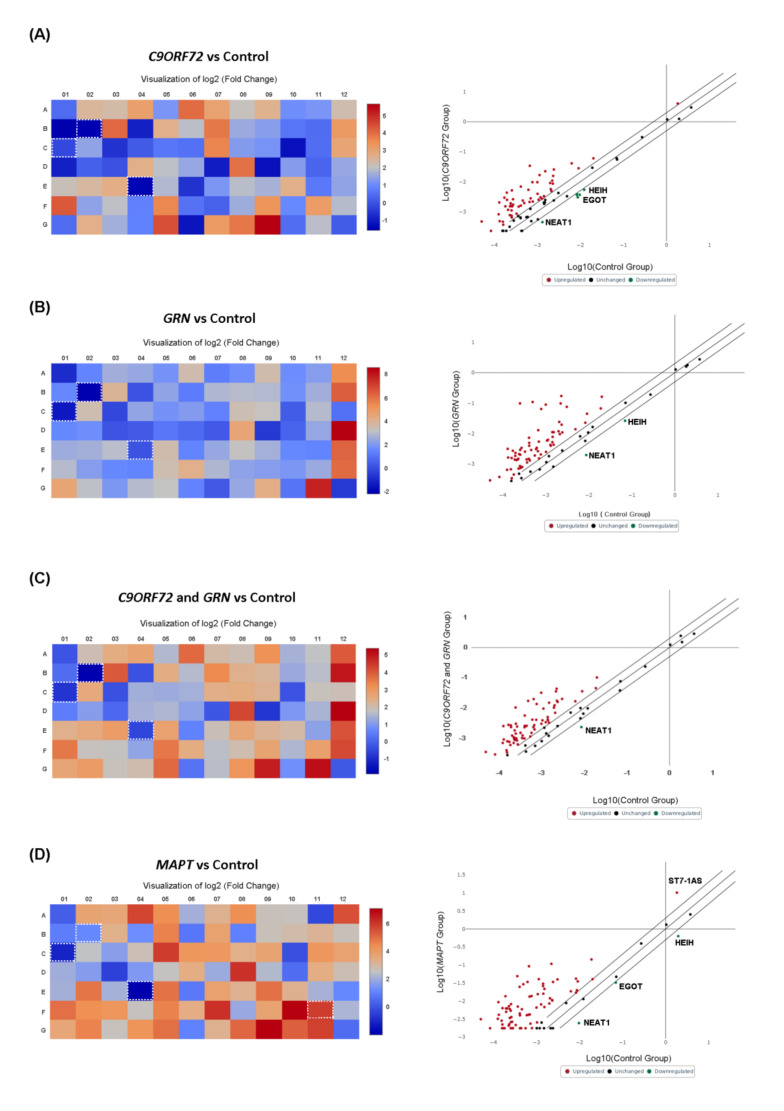
Heat Map of the expression fold changes for the 84 lncRNAs array in FTD *C9ORF72* expansion carriers (**A**); *GRN* mutation carriers (**B**); *GRN* + *C9ORF72* grouped mutation (**C**); *MAPT* mutation carriers (**D**); versus controls. Data are expressed as fold change (fold difference). Blue indicates downregulation, red upregulation. Each scatter plot compares the normalized expression of every gene on the PCR Array between the two selected groups by plotting them against one another to quickly visualize large gene expression changes. The center diagonal line indicates unchanged gene expression, while the outer diagonal lines indicate the selected fold regulation threshold. Genes with data points beyond the outer lines in the upper left and lower right corners are upregulated or downregulated, respectively, by more than the fold regulation threshold in the y-axis Group relative to the x-axis Group.

Furthermore, we tested the expression of 84 well-characterized lncRNAs in the same population. Although the statistical threshold was not reached, the results showed the generalized deregulation of lncRNA expression levels in genetic cases and in sporadic cases when compared with controls (Figure 1, panels C and D, Supplementary Tables S1 and S2). In particular, a trend toward downregulation was observed in *GRN* and *C9ORF72* patients, both when considered in the singular analysis and together according to the neuropathological protein deposition status (Figure 5 panels A-B-C, Supplementary Tables S3–S5). Conversely, an opposite trend was observed in lncRNA expression levels in *MAPT* mutation carriers, as an overall upregulation was found when compared with the controls (Figure 5, panel D, Supplementary Table S6). The same trend was found when lncRNA expression levels were investigated in sporadic patients (Figure 1, panel D, Supplementary Table S2). Moreover, some lncRNAs, including HEIH, EGOT, and NEAT1, were downregulated in all groups. Remarkably, ST7-1AS was found to be the unique lncRNA, with a trend to an upregulation in *MAPT* carriers (FC: 5.69, *p* < 0.05 FDR = 0.048, Supplementary Table S6).

## 3. Discussion

Herein an exploratory analysis of non-coding RNAs was performed for the first time considering both miRNAs and lncRNAs in a substantially well-characterized population of genetic and sporadic FTD patients in order to improve our understanding of the molecular basis underneath the clinical heterogeneity of the disease. Several dysregulations in ncRNAs, either miRNAs or lncRNAs, were observed, with different extents and trends according to the different genetic statuses. Interestingly, patients carrying mutations in *GRN* and expansions in *C9ORF72* showed a generalized downregulation in both miRNA and lncRNA expression levels. Conversely, an opposite trend was observed when *MAPT* patients were considered for the analysis. This is in accordance with the underlying proteinopathy, as *GRN* and *C9ORF72* genetic patients are associated with a TDP-43 deposition, whereas *MAPT* genetic patients are characterized by a tau protein deposition. The trend was similar also for lncRNA expression levels, as a generalized downregulation was observed in *GRN* and *C9ORF72* mutation carriers, whereas an opposite trend was shown for *MAPT* mutation carriers.

Notably, sporadic patients showed a pattern of expression of ncRNA resembling that of *MAPT* mutation carriers, although an intermediate situation would have been expected between the pathology underlying the ncRNA expression levels of *GRN* FTD and the *MAPT* one. These observations highlight the hypothesis of the existence of some common pathogenic mechanisms underneath *MAPT* genetic FTD and the sporadic form of the disease, such as altered microtubule stabilization and the increased propensity of tau self-aggregation [3].

The KEGG functional enrichment analysis revealed some interesting pathways enriched in the different FTD mutation carriers. Interestingly, RNA degradation and RNA transport pathways, as well as the phagosome and autophagy pathways, were enriched in *C9ORF72* patients and not in the *MAPT* group. As has already been ascertained, the exanucleotide repeat expansion is translated to produce dipeptide repeat proteins that are able to aggregate in patients’ brains and result in toxicity in numerous models, though the mechanisms underlying this toxicity are still poorly understood. In this scenario, altered miRNA levels targeting RNA transport and degradation genes could contribute to this aberrant pathologic molecular mechanism.

Moreover, recent evidence suggests a dysfunction in the autophagy–lysosome pathway in synergy with toxicity from *C9ORF72* RNA. In addition, C9ORF72 dipeptides have also been demonstrated to cause, by altering transport nucleoplasmic machinery, a mislocalization of TDP-43 to the cytoplasm [20,21]. 

Furthermore, delivering progranulin to neuronal lysosomes was demonstrated to protect against excitotoxicity and to be related to an increase in basal autophagy. This evidence is also supported by recent data that demonstrate that a *GRN* deficiency condition is associated with the impaired maintenance of lysosomal function and authopagy [22,23].

Conversely, some functional enrichment pathway genes seem to be specific to *MAPT* carriers. The synaptic vesicle cycle and GABAergic and cholinergic synapse pathways showed a trend to an enrichment. The genes involved in these specific pathways could likely be involved in the impairment of synaptic mediators, as recently demonstrated as occurring in genetic *MAPT* FTD patients and not in *GRN* and *C9ORF72* carriers [24]. In support of this, there is also substantial evidence of how the tau protein impacts synaptic function and how it is related to the pathological role of Beta Amyloid in synapses [25].

The message that can be deduced from the functional enrichment analysis is that determined microRNAs could drive molecular mechanisms of specific disease-associated pathways in genetic FTD.

Upon examining details of the individual dysregulations, interesting data emerged for miR-222-3p and miR-223-3p, whose expression levels were significantly upregulated in sporadic and *MAPT* mutation carriers, whereas the opposite trend was observed for *GRN* and *C9ORF72* FTD mutation carriers. MiR-223-3p has already been extensively investigated with regard to neurodegenerative diseases, where it was found to have a neuroprotective effect and to be a neurodegenerative biomarker that is able to discriminate AD patients from controls and from PD patients [18,26].

In addition, miR-155-3p, miR-16-5p, and miR27a-3p were already found to be dysregulated in other neurodegenerative diseases, such as sporadic ALS, suggesting some possible common overlapping mechanisms [27,28].

Among the lncRNAs found to be dysregulated, NEAT1 is worthy of particular interest since it has already been investigated with regard to the proposed pathogenic mechanism underneath the *C9ORF72* pathology. It has been recently demonstrated that the expression of NEAT1 is deregulated in FTD [29]. Contextually, it has also been observed that FTLD-TDP cases showed an increase in TDP-43 binding through lncRNA NEAT1 in the cortex. Furthermore, the overexpression of lncRNA NEAT1 ameliorates the toxicity of TDP-43 in *Drosophila* and in TDP-43 protein disease yeast models, which can lead to the conclusion that NEAT1 upregulation may be protective against TDP-43 proteinopathies affecting the brain [30]. This body of evidence likely supports the challenging perspective that FTD might be treated by increasing the expression of lncRNA NEAT1 in the central nervous system.

In conclusion, we showed that there is a clear distinctive pattern of dysregulation among ncRNAs between the mutations associated with the TDP-43 pathology and the mutations associated with the tau pathology. Some of these dysregulations have also been observed in other neurodegenerative diseases, such as Alzheimer’s disease, suggesting the role of ncRNA in driving neurodegenerative processes [31].

However, only a few ncRNAs reached the statistical threshold, most likely due to the small number of patients considered for each group. Therefore, further analysis using a larger population is required to draw definitive conclusions and confirm our preliminary results.

## 4. Materials and Methods

### 4.1. Population

Thirty-two patients with genetic and sporadic FTD were recruited at the Alzheimer’s Unit of Fondazione Ca’ Granda, IRCCS Ospedale Maggiore Policlinico (Milan) and Fondazione IRCCS Istituto Neurologico Carlo Besta (Milan). The FTD population was composed of 8 sporadic FTD patients, 8 *GRN* mutation carriers, 8 *MAPT* mutation carriers, and 8 *C9ORF72* expansion carriers. All patients underwent a standard battery of examinations, including medical history, a physical and neurological examination, screening laboratory tests, a neurocognitive evaluation, and imaging. The Clinical Dementia Rating (CDR), the Mini-Mental State Examination (MMSE), the Frontal Assessment Battery (FAB), the Wisconsin Card Sorting Test (WCST), and the Tower of London test assessed the degree of cognitive impairment. The presence of significant vascular brain damage was excluded (Hachinski Ischemic Score < 4). FTD patients were diagnosed according to current consensus criteria [32]. The control group consisted of twelve non-demented volunteers matched for ethnic background and age and without memory and psychobehavioural dysfunctions (MMSE ≥ 28).

This study was approved by the Institutional Review Board of the Fondazione Ca’ Granda, IRCCS Ospedale Maggiore Policlinico (Milan, Italy). All patients and/or their caregivers provided their written informed consent.

The characteristics of patients and controls are summarized in Table 1.

### 4.2. Serum Samples Collection and Total RNA Isolation

Whole-blood samples were allowed to sit at room temperature for at least 30 min after collection. Clot separation was achieved with centrifugation at 1000–1300× *g* at room temperature for 15–20 min. The serum samples were collected in aliquots of 500µL into cryogenic vials and stored at −80 °C until analysis. Afterward, the serum samples were thawed on ice and lysed with an equal volume of 2X Denaturing Solution (Ambion, Austin, TX, USA). To allow sample-to-sample normalization, synthetic *C. elegans* miRNA cel-miR-39 (synthetic RNA oligonucleotides synthetized by Qiagen, Hilden, Germany) was added (as a mixture of 25 fmol of each oligonucleotide in 5 mL total volume) to each denatured sample. The RNA was isolated using an MiRNeasy serum/plasma Qiagen kit according to the instruction of the manufacturer.

### 4.3. Screening of miRNAs and lncRNAs in Human Serum Samples

**Figure 6:**
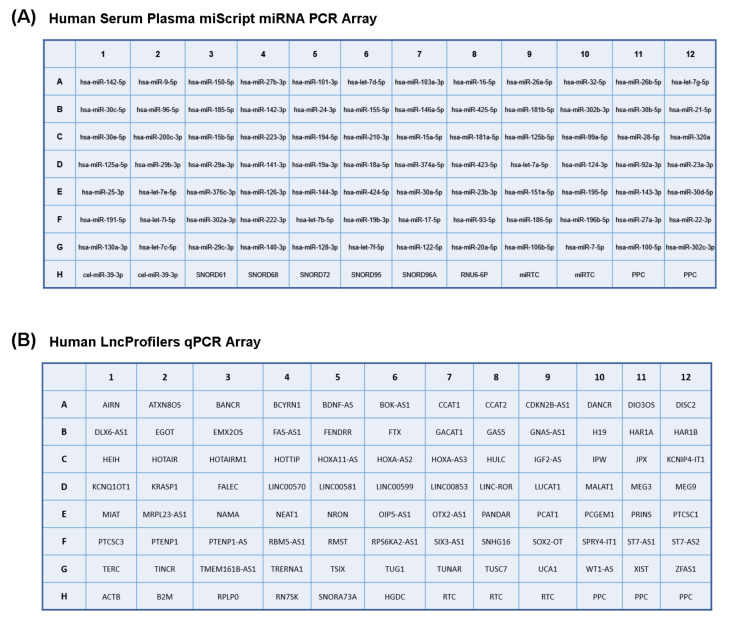
(**A**) plate layout for Human Serum & Plasma miScript PCR Array. Cel-miR-39-3p are spike-in controls SNORD61, SNORD68, SNORD72, SNORD95, SNORD96A, RNU6-6P; housekeeping genes miRTC are controls for retrotranscription, PPC positive controls for PCR. (**B**): Plate layout for Human LncProfilers qPCR Array. Endogenous reference RNAs for the normalization: ACTB, B2M, RPLP0, RN7SK, SNORA37A, as well as control for human genomic DNA contamination, reverse transcription controls, and positive PCR controls.

miRNAs and lncRNAs were retrotranscribed either with a miScript II RT kit (Qiagen) or by using reverse transcriptase and random primers according to the instructions of the manufacturer. For Real-Time PCR experiments, we used the Human Serum & Plasma miScript miRNA PCR Array using a 7500 FAST system. The miScript miRNA PCR Array profiles the expression of the 84 most abundantly expressed and best-characterized miRNA sequences in the serum. This array also includes a spike-in control element (*cel-miR-39*) and short non-coding RNAs (SNORD61, SNORD68, SNORD72, SNORD95, SNORD96A and RNU6B-2) for the proper normalization of the data, miRNA reverse transcription control, and a positive PCR control (Figure 1). Human LncProfilers qPCR Array kit profiles the 84 well-characterized lncRNAs and includes five endogenous reference RNAs for the normalization (ACTB, B2M, RPLP0, RN7SK, SNORA37A), as well as controls for human genomic DNA contamination, reverse transcription controls, and positive PCR controls according to the layout depicted in Figure 6. 

### 4.4. Statistical Analysis

The PCR Array data analysis was based on the ΔΔCt method, with the normalization of the raw data to housekeeping genes and using the software available at https://dataanalysis.qiagen.com/mirna/arrayanalysis.php, accessed on 20 July 2022 whereas analysis for the lncRNA was performed with the proper software at https://dataanalysis2.qiagen.com/lncRNA, accessed on 21 July 2022.

The *p*-values were calculated based on a Student’s *t*-test of the replicate 2^−ΔCt^ values for each gene in the control and FTD groups. The best hits were chosen based on their statistical significance (*p* < 0.050) [33]. The results are further expressed as fold changes (FC), and defined as the normalized miRNA expression in each test sample divided by the normalized miRNA expression in the control sample.

Benjamin–Hochberg’s false discovery rate (FDR) correction was used to correct multiple comparisons (*p* < 0.05).

### 4.5. Target Prediction and Pathway Enrichment Analysis

The MiRNet (https://www.mirnet.ca/miRNet/home.xhtml, accessed on 12 September 2022) web tool was used to provide visual exploration and functional interpretation of miRNA-target interaction network and a pathways enrichment analysis [34]. Functional enrichment analysis was performed using the KEGG database with two different algorithms implemented in the miRNet tool: hypergeometric tests and empirical sampling, as recently proposed [35]. The targets were also associated with specific diseases by using DisGeNET (https://www.disgenet.org/home/, accessed on 13 September 2022), implemented in the miRNet tool.

## Figures and Tables

**Table 1:** Characteristics of patients and controls.

	FTD	Controls
Number of subjects	32	12
Gender (M:F)	20:12	7:5
Mean age, years ± SD	72.7 ± 6.2	73.2 ± 1.99
Mean age at onset, years ± SD	66.50 ± 0.44	
Mean disease duration, years ± SD	3.01 ± 0.23	

SD, Standard Deviation.

## Supplementary Materials

The following supporting information can be downloaded at: https://www.mdpi.com/article/10.3390/ijms232314723/s1.
